# Gender equity in planning, development and management of human resources for health: a scoping review

**DOI:** 10.1186/s12960-019-0391-3

**Published:** 2019-07-11

**Authors:** Nour El Arnaout, Rana F. Chehab, Bayan Rafii, Mohamad Alameddine

**Affiliations:** 1 Global Health Institute, American University of Beirut, Riad El Solh, Beirut, 1107 2020 Lebanon; 2 Department of Nutrition Science, College of Health and Human Sciences, Purdue University, West Lafayette, IN 47907 USA; 3 Department of Health Management and Policy, Faculty of Health Sciences, American University of Beirut, Riad El Solh, Beirut, 1107 2020 Lebanon

**Keywords:** Gender, Equity, Human Resources, Health, Management, Planning, Development

## Abstract

**Background:**

Gender equity remains a challenge across various labor markets with the health market being no exception. Despite the increased influx of women into health professions, horizontal and vertical occupational gender inequities persist.

**Main body:**

The objective of this scoping review is to map the studies on gender equity in healthcare systems in terms of workforce planning, development, and management, as well as to identify the barriers and facilitators for integrating gender equity into healthcare systems. We reviewed the literature on the topic using nine electronic and two grey literature databases with the search strategy combining medical subheadings and keywords for each of the following four concepts of interest: “gender equity,” “human resources for health,” “healthcare setting,” and “management processes.” The scoping review included studies focusing on the examination of gender equity at the level of the health workforce. Out of 20,242 studies identified through the database search, the full text of 367 articles was assessed for eligibility and 110 were included in the qualitative analysis. The data of those studies was abstracted and analyzed into themes.

Results do not only reveal a global dearth of studies focused on this important topic, but also the concentration of such studies in a few countries around the globe, mainly in North America and Europe. Four out of each five studies included in this review focused on physicians, followed by nurses (14%). In terms of design, an overwhelming majority of studies utilized quantitative designs (75%), followed by qualitative designs and database analyses. Studies were categorized into four pre-determined main themes: facilitators and barriers, workforce planning, HRH management, and HRH development.

**Conclusion:**

Future research is needed to better understand poorly covered sub-themes such as mentorship, professional development, and training, as well as recruitment and retention among others. It is also equally needed to fill in the gaps in professional groups, study type, methodology, and region. While the review unearthed a number of well-studied themes, significant aspects of the topic remain untapped especially in developing countries and at the level of health professionals other than physicians.

**Electronic supplementary material:**

The online version of this article (10.1186/s12960-019-0391-3) contains supplementary material, which is available to authorized users.

## Background

Gender equity and women’s empowerment have been set by the United Nations as unique goals on the 2030 global agenda for sustainable development [1]. Although progress has been made towards achieving this goal and despite that women participation in the workforce has been growing rapidly [2], women remain under-represented occupying less than a third of leadership and management positions [3]. In the labor market, there has been a wage gap between men and women for decades [4], with women worldwide earning 23% less than men [5]. However, women carry out around three times more unpaid household work, child care, and elderly care compared to men [3]. In addition, reports show that in the past 10 years, the global economic gender gap narrowed by only 3%, with a current global economic participation and opportunity gap still standing at around 40% [6]. In light of the slow progress towards gender equity, the 2017 Gender Gap Report forecasts that it would take some countries 100 years to close their gender gaps, with the most challenging gaps in the economic and health spheres [7]. Factors contributing to this gender gap are many; these include unequal career opportunities, gender insensitive institutional policies, gender discrimination in recruitment, gender inequitable staffing, increased risk of violence against women workers, poor working conditions, lack of development and mentorship, among others [8–10].

Human resources for health (HRH) including physicians, nurses, and pharmacists, among many other professionals are no exception to the global trends of gender inequity in the workplace. Despite the increased influx of women into these health professions [11, 12], horizontal (refers to the number of individuals of each gender present at each occupation) and vertical (refers to male domination of highest ranked jobs) occupational gender segregation persist [13]. Across the different health professions, leadership positions occupied by women are scarce, reflecting gender inequity in regard to career advancement and attainment of decision-making positions [14–16]. Gender disparities are also noticed in other management aspects of HRH where women are reported to earn less than men, underlining consequently an existing compensation gap [17–19]. Similarly, studies show that women HRH often have lower likelihood of promotion and slower career advancement compared to men HRH in the same field [15, 16, 20].

Several theories lay the ground for the existing gender difference in the workplace, attributing the gap to different reasons. For example, the Human Capital theory attributes the inequality between women and men in the workplace to the differences in experience and skills [21, 22]. This theory further suggests that women devote more time to childcare, elderly care, and household work and thus prefer part-time positions, have more interrupted careers, and take different educational paths than men—all of which are considered an underinvestment in human capital [21]. For example, a study by Witter et al. on gendered health workforce suggests that in-service training was more difficult for women health workers to pursue given that it required more time away from home [10], despite that these trainings are often linked to promotional opportunities. Similarly, the Role Conflict theory highlights the difficulties faced by working women in combining and simultaneously succeeding in professional and family responsibilities [13]. On the other hand, the Gender Stratification theory attributes the gender disparities in the workplace to the stereotypical assumptions and discriminatory approaches of the recruiters and managers who often doubt the capabilities of female human resources and their commitment to work [21]. For instance, Newman et al. suggest that gender segregation and stratification exists at the level of the health workforce through the distinction between occupations from the one hand, and the relations between occupations on the other hand, stating that this segregation could be mitigated by the adoption of equal opportunity policies at the institutional level [23]. Comparably, the Institutional theory goes beyond blaming the individual female employee and attributes the gender inequity in the workplace to the set of organizational and structural policies, as well as the organizational culture of the employing institution [4]. The institutional theorists identify policies such as inflexible working hours, unavailability of convenient child care arrangements, and absence of job sharing as impediments towards gender equitable work environment [4].

The existing gender disparities with regard to career advancement across different professions have been translated theoretically by the leaky pipeline theory [24]. The latter states that the proportion of women decreases disproportionately at every stage of the career ladder. This underlines remarkable gender inequality in human resources (HR) retention process [16, 25]. Similarly, the “glass ceiling” metaphor has been used in the literature to describe the limited attainment and scarcity of women in advanced, highest-paying, decision-making positions, despite their increased entry to fields [16, 26]. This observation is often attributed to the difference in the access of HRH of both genders to equal opportunities of mentorship, career development, networking, and role models, amid current work environments and organizational cultures that favor men and seem to place women at a disadvantage [27].

Despite the various theories and large number of studies focusing on the existing gender gap among HRH across different healthcare professions, to the best knowledge of the authors a review that comprehensively presents the wide range of these gaps is missing in the literature. There remains a need to prioritize areas to be addressed based on evidence to set the agenda for moving forward effectively in enhancing gender equity in the planning, development, and management of HRH.

The objective of this scoping review is to map the studies on gender equity in the health workforce in terms of planning, development, and management, as well as the barriers and facilitators for integrating gender equity into the health workforce.

## Main text

### Methods

#### Protocol and registration

The protocol for this scoping review, which followed the Arksey and O’Malley framework [28], was registered in the PROSPERO prospective register of systematic reviews under registration number CRD42016042372.

#### Search strategy

A systematic search strategy (Additional file 1) combined both medical subheadings (MeSH) and keywords for each of the following four concepts of interest: “gender equity” (including gender sensitivity, gender equality, gender discrimination…), “human resources for health” (including health care provider, doctor, nurse…), “healthcare setting” (including hospital, health center, medical school…), and “management processes” (including planning, development, recruitment, retention…). The search strategy was run on the following seven electronic databases: PubMed, MEDLINE, EMBASE, CINAHL, Sociological Abstracts, Scopus, and Cochrane Library; as well as two grey literature databases: ProQuest Dissertations and Theses, and Open Grey.

#### Eligibility criteria

The scoping review included the following types of primary studies: randomized and non-randomized trials, case-control, cohort, case studies, cross-sectional, and qualitative. Commentaries, opinion pieces, and reviews were excluded. Only studies that have abstracts and full texts accessible in English and published between January 1, 1996 to July 1, 2017 were included. The inclusion of target population was limited to HRH working in all healthcare organizations (private, public, etc.), including physicians (e.g., surgeons and dentists), nurses, pharmacists, nutritionists, healthcare administration and management, and other healthcare workers. The definition of HRH adopted in our review is that of the World Health Organization in which HRH are defined as individuals engaged in actions whose primary intent is to enhance health. These human resources include clinical staff such as physicians, nurses, pharmacists and dentists, as well as management and support staff.

#### Screening and selection process

The results of the search strategy were exported to Endnote and duplicates were removed. A two-stage selection process was conducted: the title and abstract screening stage, and the full-text screening stage. In the first stage, the titles and abstracts of the identified citations were screened for potential eligibility by two reviewers in duplicate and independently. In the second stage, the team of two reviewers screened the full texts of the studies for eligibility. Disagreements in inclusion during both the title and abstracts as well as the full-text screening were resolved through discussion, and by the help of a third reviewer, as needed. Agreement level between reviewers was calculated using the kappa statistic. Reference lists of all included studies were screened to identify additional citations for potential eligibility of inclusion.

#### Data abstraction process

The research team developed a data abstraction table that included the following items: publication date of the study, study design, country and setting where the study was conducted, population subtype group, data collection tool, and themes addressed in each study.

#### Data synthesis

Given the nature of the data collected, extracted results were analyzed thematically. Sub-themes in each study were grouped into pre-determined thematical categories guided by the review’s research questions; these are (1) barriers/ facilitators to integration of gender equity, (2) workforce planning, (3) HRH management, and (4) HRH development. A numerical descriptive summary of the studies included in this scoping review was presented. Study designs, data collection tools, countries where the studies were conducted, publication years, study populations, and the setting were reflected in tabular and graphical representations.

### Results

**Fig. 1:**
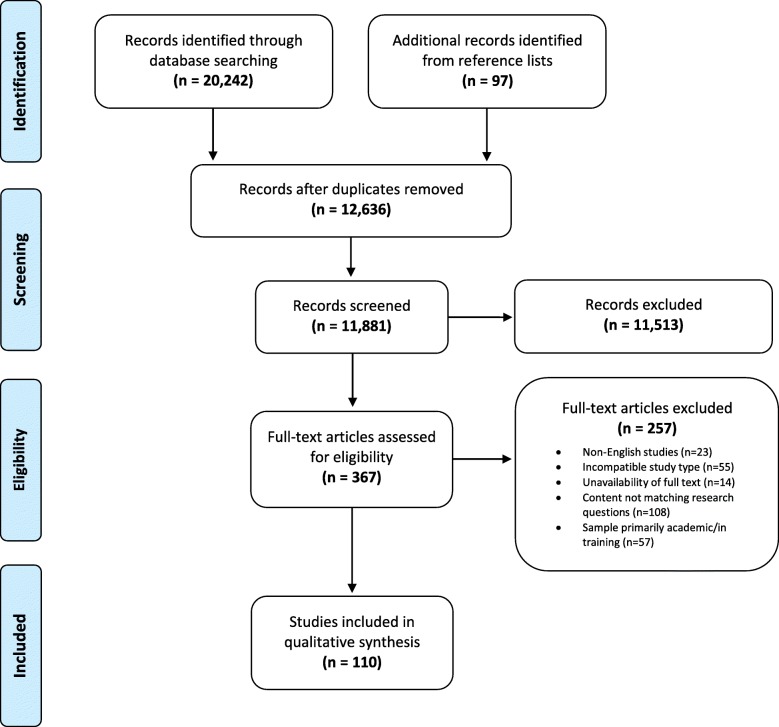
The PRISMA flow diagram for the selection process of studies and reasons for exclusion

The selection process is shown in Fig. 1. Out of 20,242 articles obtained from the systematic search on electronic databases, and the 97 articles identified from reference lists, a total of 11,881 studies were eligible for title and abstract screening, after removal of duplicates. Three hundred sixty-seven studies were selected for full-text screening and 110 articles were included in the analysis of this review. Agreement between reviewers was calculated using the kappa statistic which was found to be 0.65, suggesting substantial agreement between reviewers [29].

#### Characteristics of the included studies

The characteristics of the included studies are presented in Additional file 2.

#### Study design and data collection tools

Out of the 110 included studies, the majority adopted a quantitative approach (75%), using surveys/questionnaires as an exclusive data collection tools. Qualitative studies were used to a lesser extent (9%) and reported findings using semi-structured interviews, focus groups, and observation. Only few studies adopted a mixed methods approach (6%), combining qualitative data collection tools with close-ended questionnaires. In 9% of the studies, information was collected from existing databases or through an online search of government or organization reports and websites. None of the included studies employed an interventional approach such as randomized trials (Additional file 3).

Seven percent of the studies employed secondary data analysis of existing databases to study aspects like recruitment and salary. Two percent relied on online search of websites and reports to compile the relevant data (e.g., male to female ratio of top leadership).

#### Regions and countries of studies

**Fig. 2:**
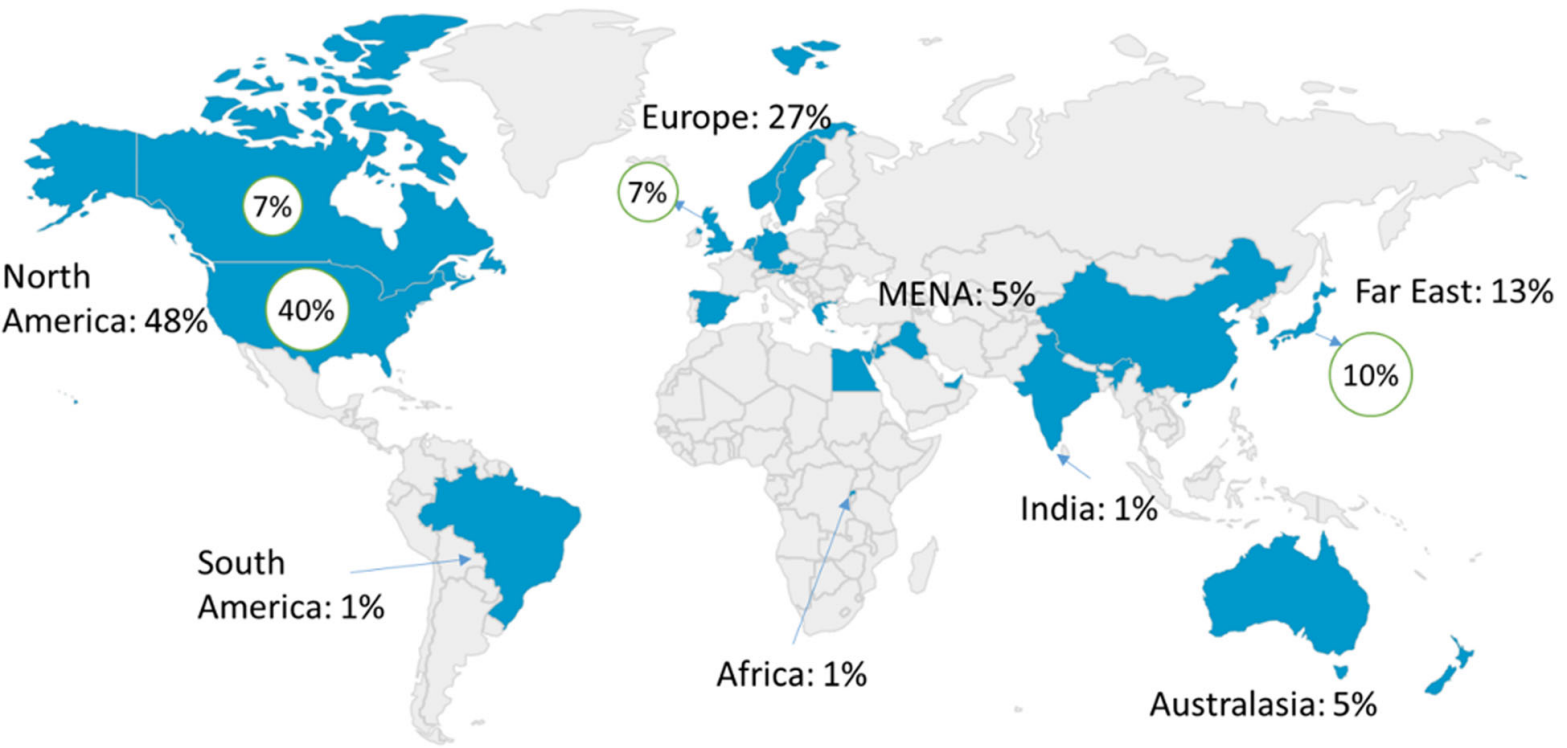
Proportional distribution of included studies by world region. Four countries with high publication yield (United States of America, Canada, United Kingdom, and Japan) were also highlighted in a green circle (their contributions are part of the regional average).

The majority of papers were published in Western countries (80%), originating mainly from North America (48%), followed by Europe (27%) then Australia (5%) (Fig. 2). Out of the non-Western regions, the Far East (13%) had the greatest contribution followed by the Middle East and North Africa (MENA) region (5%). While the number of studies came mostly from the Western Region, the number of countries represented by studies from that region was similar to the number of countries in the non-Western region. Countries with the greatest number of studies were the United States America (40%), Japan (10%), Canada (7%), and the United Kingdom (7%) (Fig. 2).

#### Studies per year

**Fig. 3:**
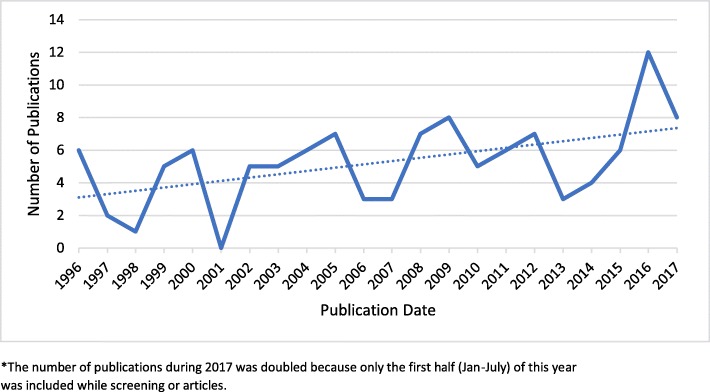
Number of publications per year (solid line) from year 1996 to 2017* and linear trend-line (dotted)

There was a gradual increase in the number of studies over the years, with a median number of studies of 5.5 per year (Fig. 3). The annual number of publications fluctuated with some obvious peaks in 2009 and 2016. However, there seems to be an increasing trend in the number of publications over the years, indicating heightened attention to an ongoing challenge.

#### Professional groups and settings

The included studies covered a range of HRH with 5% of the included studies covering all health workers without particular focus on any single professional group. Overall, the majority of the studies addressed issues related to physicians (79%), followed by nurses, to a lesser extent (14%). Among physicians, the most common specialists were surgeons and gynecologists/obstetricians covering for 20% and 14% of physicians; respectively. Pharmacists (2%) were the least targeted HRH when discussing gender equity issues.

Half of the studies were not specific to any health setting (54%) as samples were drawn from the general HRH population with no specification of setting. The other half of the studies focused on populations sampled from a hospital setting (24%), health and medical centers (11%), medical schools (6%), clinics (5%), and a hospice (1%).

#### Themes of the studies

Guided by the research questions, the included studies were categorized into four main themes: (1) barriers/facilitators to integration of gender equity, (2) workforce planning, (3) HRH management, and (4) HRH development. These themes were based on human resources management framework, applicable to healthcare settings [30]. Barriers and facilitators are defined in this review as factors that impede or enhance the applicability of gender equitable practices relating to the HRH management processes including planning, development, and others. Workforce planning is defined as the right level and mix of HRH are available to deliver needed services to a target population [31]. HRH management reflects practices applied in an organization to manage its HRH [32], while HRH development is a process of optimizing the production and utilization of the HRH; included in the scope of HRH development are staffing, training and education, performance management, and working conditions [33].

**Fig. 4:**
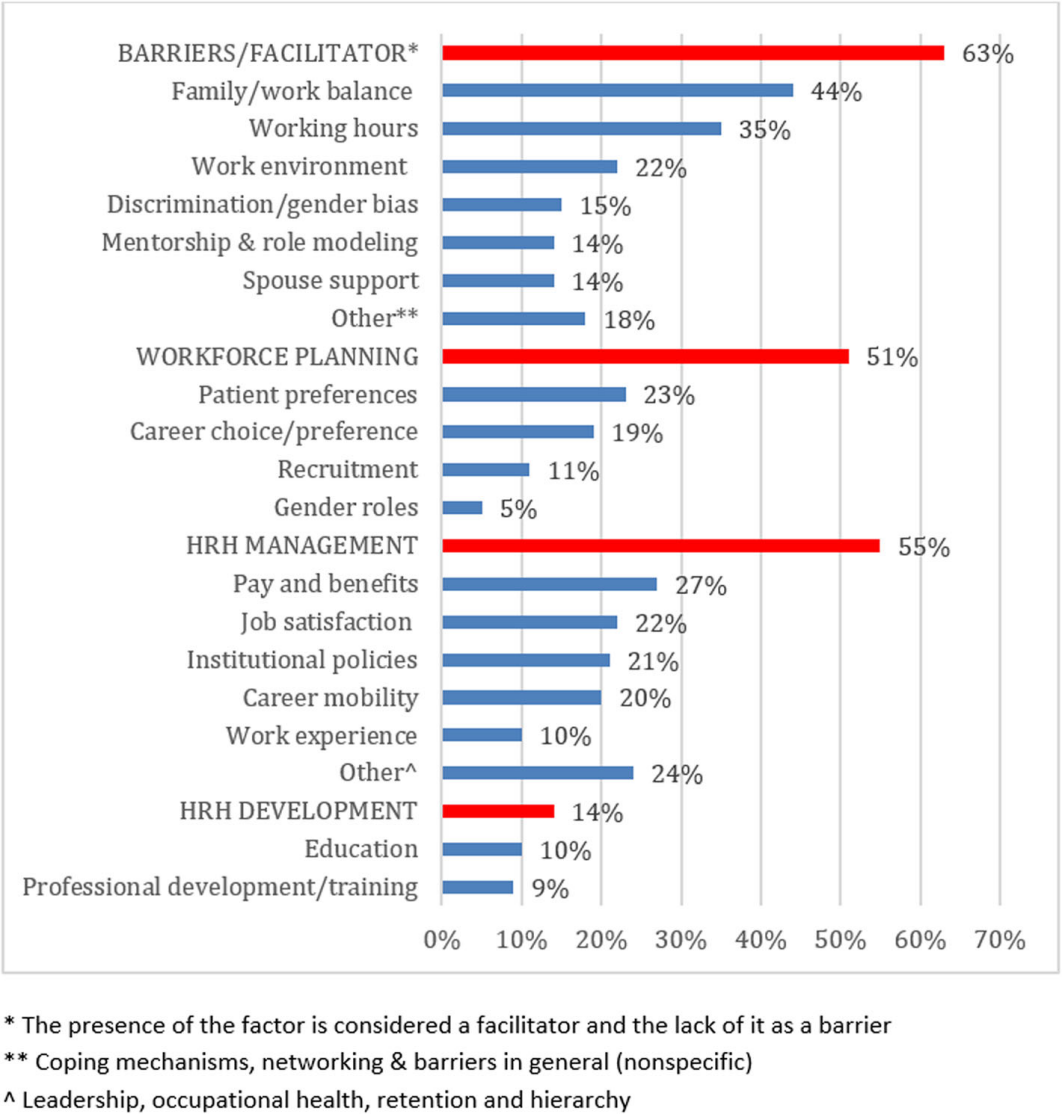
Themes (capitalized) and sub-themes emerging from the analysis of included manuscripts. Note that themes are not mutually exclusive, and percentage is calculated out of the total number of included studies (110)

Themes were not mutually exclusive, with some studies covering multiple themes. A total of 24 sub-themes were extracted from the studies and were organized under the four main themes (Fig. 4).

The first theme was Barriers/Facilitators for gender equity which was addressed in 63% of studies (69 studies) covering nine sub-themes. The top sub-themes included family/work balance (44%), working hours (35%), work environment (22%), and discrimination/gender bias (15%). Workforce planning was the second overarching theme covered in 51% of studies (56 studies) and included four sub-themes, specifically patient preferences (23%), career choice/preference (18%), recruitment (11%), and gender roles (5%). HRH Management was the third main theme which was covered in 55% of studies and included nine sub-themes. Pay and benefits (27%) was most commonly studied followed by job satisfaction (22%), institutional policies (21%), and career mobility (20%). HRH Development was the fourth main theme which was discussed in 14% of the studies (15 studies) covering two sub-themes: education (11%) and training (10%).

### Discussion

This study provides an initial attempt to comprehensively review published studies on gender equity in healthcare systems in terms of workforce planning, development, and management. Most of the included studies stemmed from North America and Europe. Four out of each five studies included in this review focused on physicians, followed by nurses (14%). In terms of design, an overwhelming majority of studies utilized quantitative designs (75%), followed by qualitative designs and database analyses. Studies focused on four main themes: Facilitators and barriers, workforce planning, HRH management, and HRH development.

Results do not only reveal a global dearth of studies focused on this important topic, but also the concentration of such studies in a few countries around the globe, mainly in North America and Europe. This is interesting taking into consideration that the countries publishing most about the topic are the ones that have relatively better gender equity records and practices [25]. Other countries, especially the ones ranking low on the gender equality index are thus encouraged to invest more in researching this important topic to provide the evidence needed to guide the formulation of equitable workforce policies and practices.

Another important finding of this review relates to the focus of studies examining equity in planning, management and development of HRH on physicians, followed by nurses with very few studies addressing other healthcare professionals. While ensuring equity among physicians is necessary, it is not sufficient taking into consideration that physicians are not the largest professional group in the health sector and a multidisciplinary healthcare team engages tens of healthcare professionals. Nurses compose a large percentage of HRH and are known to face major gender disparities such as harassment, discrimination/gender bias, inflexible working hours, and gender inequity in career mobility [34–36]. The effective management of HRH requires expanded attention to researching gender equity in other health professional groups, especially that many health professions are known to engage a female majority constituency. Public and private funding agencies are strongly encouraged to support research studies that examine the equitable planning, management and development of various types of health professionals so that their voice is integrated in the formulation of gender equitable HRH policies and practices.

This scoping review demonstrated how multifaceted the topic of gender equity in HRH is and that there are many aspects to take into consideration when developing policies to improve gender equity in health management systems. Despite the large number of identified themes and sub-themes, studies mostly focused on a small subset of themes such as family-work balance, working hours, and pay and benefits, while other sub-themes were much less investigated; these include mentorship, professional development and training, recruitment, retention, work experience, and spouse support among others. When considering the existing theories on gender equity, many remain poorly understood for HRH. Theories focusing on family-work balance such as the Role Conflict Theory and related parts of the Human Capital Theory are well supported by the literature. On the other hand, aspects in the Human Capital Theory such as career choice/preference, work experience and skill sets are not well understood. Furthermore, only around 15% of the studies included in our review contribute to our understanding of the Gender Stratification Theory which focuses on discrimination/gender bias and recruitment. Similarly, only a small percentage of the literature give insight into the Institutional Theory where institutional policies, hierarchy/organizational structure, and work environment are highlighted as major reasons for gender inequity. Finally, our understanding of the Leaky Pipeline Theory is limited with only 20% of the studies focusing on career mobility and less than 10% on topics of leadership, retention, and hierarchy/organizational structure.

With respect to study methodology, most studies focused on quantitative methods (75%) whereas qualitative and mixed method studies only consisted of 15% of the literature. This is unfortunate as quantitative and mixed methods studies can often more accurately characterize the enablers and barriers for the integration of gender equity in HRH planning, management and development than can be done with quantitative studies. Finally, no intervention and evaluation studies, including return on investment studies, were found in the literature. These studies are important to generate evidence-based policy and practice recommendations.

In terms of thematic focus of the reviewed studies, enabling a family/work balance and reasonable work hours emerged as the most prominent themes enabling or hindering gender equity of the HRH workforce depending on how well the planning and management of the workforce take this into consideration. Attention to these sub-themes is instrumental in enhancing workforce participation and integration of professional working females [15, 37] who constitute the majority of the health workforce [38]. Ensuring supportive and inclusive work environment is also instrumental for gender equity and is directly correlated with enhancing job satisfaction and retention [39]. Furthermore, safeguarding gender equity in pay and benefits is also a key theme that is well documented in literature with the gap closing in some countries and regretfully widening in others [40]. Attention to this important sub-theme is important to enhancing gender equity in the health sector. Last but not least, ensuring equitable access to education and professional development opportunities is a significant factor that needs to be safeguarded taking, into consideration the large body of literature that correlates education and training with satisfaction and retention in the sector [41, 42].

Future research is needed to better understand poorly covered themes as well as fill in the gaps in professional groups, study methodology and type, and regions. Furthermore, several reviews can focus on specific themes or groups of themes and summarize the literature of these topics to guide future policy. Moreover, governments, funding agencies, and foundations are encouraged to fund research programs examining the integration of gender equity in the planning, organization, and management of the healthcare field. Preference should be offered to studies that evaluate programs and interventions using qualitative or mixed methods. Particular attention should be offered to fostering such research programs in Africa, South America and the MENA region and the non-Western region in general. East-West and North-South collaborations may be beneficial in building capacity and contextualizing experiences. There is also a need to fund, implement, and evaluate a thorough examination of the compensation gap between HRH in the healthcare sectors in all countries. The legal and regulatory frameworks need to be modified to enable the closing of identified gaps. Work policies and procedures need to be re-examined to offer employment flexibility to the professional female workforce and their partners, especially in the childbearing age, enabling them to maintain work-life balance and retaining them in the active labor market.

A number of limitations in this study are noteworthy. First, the sample selection criteria excluded studies with sample populations primarily in training or academia. While this maintained the focus of the study, it may have buffered the findings on the education and training theme. It is recommended that future reviews focus on examining gender equity in training and academia. Second, non-English speaking countries may have had more studies on this topic but published in their own language. This may partially explain the low number of non-Western literature. For researchers studying a non-English speaking country, it is recommended to review the reference lists of the studies of this country included in this review to find non-English literature on the subject. Finally, studies done outside of the timeframe (January 1, 1996 and January 7, 2017) were not captured.

## Conclusion

Although this scoping review underlined the efforts of researchers to investigate different aspects of gender equity in planning, development, and management of HRH at the systems level, significant other aspects of the topic remain untapped especially in developing countries and at the level of health professionals other than physicians.

## Additional files

Additional file 1: Search stratgey of the study. (DOCX 34 kb)

Additional file 2: Table of the 110 included studies with their descriptive categories (country, region, professional group, setting, study design, and tool) and themes. (XLSX 45 kb)

Additional file 3: Description of included studies; alignment of gender theories with used search strategy terms and results sub-themes. (PNG 136 kb)

## Data Availability

All data generated or analyzed during this study are included in this published article and its supplementary information files.

## Abbreviations

HR: Human resources
HRH: Human resources for health
MeSH: Medical subheadings
