# Supplementary material for: Gender equity in planning, development and management of human resources for health: a scoping review
Source: Hum Resour Health. 2019 Jul 11;17:52. doi: 10.1186/s12960-019-0391-3 (PMC6625080; doi:10.1186/s12960-019-0391-3)
Supplement: Supplementary file 1 — Search stratgey of the study. (DOCX 34 kb) [file 12960_2019_391_MOESM1_ESM.docx]

Search Strategies

**Gender Equity in Health Human Resources Planning, Development and Management: A Scoping Review**

Contents

[Medline 1](#_Toc511040390)

[Pubmed 3](#_Toc511040391)

[Scopus 7](#_Toc511040392)

[CINAHL 9](#_Toc511040393)

[Sociological Abstracts: 11](#_Toc511040394)

[Cochrane 14](#_Toc511040395)

[Embase 16](#_Toc511040396)

[Grey literature 17](#_Toc511040397)

[OpenGrey 17](#_Toc511040398)

[ProQuest Dissertation and Theses 17](#_Toc511040399)

# Medline

Database: Ovid MEDLINE(R) without Revisions <1996 to February Week 2 2016> (later rerun to include 07-02-2016 to 01-07-2017)

Search Strategy:

--------------------------------------------------------------------------------

1 Sex Factors/

2 Sexism/

3 sexism.tw.

4 Feminism/

5 feminism.tw.

6 ((gender or sex) adj2 (sensitiv* or equit* or inequit* or equalit* or inequalit* or budget* or mainstream* or discriminat* or stereotyp* or bias or issue* or factor* or variab* or difference* or conflict* or diversit* or analysis or relation*)).tw.

7 or/1-6

8 education/ or curriculum/ or competency-based education/ or interdisciplinary studies/ or problem-based learning/ or education, predental/ or education, premedical/ or education, professional/ or exp education, continuing/ or exp education, dental/ or exp education, graduate/ or exp education, medical/ or exp education, nursing/ or exp education, pharmacy/ or education, public health professional/ or exp internship, nonmedical/

9 Learning/

10 Preceptorship/

11 inservice training/ or staff development/

12 personnel management/ or career mobility/ or employee incentive plans/ or personnel administration, hospital/ or personnel selection/ or "personnel staffing and scheduling"/ or personnel turnover/ or physician incentive plans/ or workload/

13 (education or learning or preceptorship* or mentorship* or residenc* or internship* or training* or orientation* or development or recruitment* or retention* or retaining or hiring or planning or management or selection or staffing or scheduling or motivation or promotion or performance).tw.

14 (turnover or (turn adj over) or (career adj mobility) or workload* or (work adj load*)).tw.

15 income/ or "salaries and fringe benefits"/ or family leave/ or sick leave/

16 (salar* or fringe* or incentive* or income* or wage* or (pay adj equit*) or ((sick or matern*) adj leave*) or absenteeism).tw.

17 Needs Assessment/

18 (need* adj assessment*).tw.

19 or/8-18

20 exp schools, health occupations/ or universities/

21 health facilities/ or exp academic medical centers/ or exp health facility administration/ or exp hospital administration/ or exp hospital units/ or exp hospitals/ or laboratories, hospital/ or pharmacies/ (280274)

22 (((medical or health or academic) adj2 (center* or centre or school* or facilit*)) or hospital*).tw.

23 or/20-22

24 Health Manpower/

25 exp Health Personnel/

26 exp Students, Health Occupations/

27 (human adj2 (capital or resource*)).tw.

28 (worker* or (work adj force) or workforce or (man adj power) or manpower or personnel or staff).tw.

29 ((health adj care adj provider*) or doctor* or practitioner* or physician* or clinician* or staff or specialist* or surgeon* or nurse* or pharmacist* or dentist* or dieti?ian* or nutritionist* or (medical adj lab adj specialist*) or radiologist* or (physical adj therapist*) or manager* or administrator* or leader* or student* or trainee* or intern*).tw.

30 or/24-29

31 7 and 19 and 23 and 30

***************************

# Pubmed

| **Search** | **Query** |
| --- | --- |
| **#27** | **Search #5 AND #18 AND #22 AND #26** |
| **#26** | **Search #23 OR #24 OR #25** |
| #25 | Search gender sensitivity[tw] OR gender sensitive[tw] OR gender equitable[tw] OR gender equity[tw] OR gender inequitable[tw] OR gender inequities[tw] OR gender inequity[tw] OR gender equalitarian[tw] OR gender equality[tw] OR gender inequalities[tw] OR gender inequality[tw] OR gender budgeting[tw] OR gender mainstreaming[tw] OR gender discriminated[tw] OR gender discriminating[tw] OR gender discrimination[tw] OR gender discriminations[tw] OR gender discriminative[tw] OR gender discriminatory[tw] OR gender stereotype[tw] OR gender stereotyped[tw] OR gender stereotypes[tw] OR gender stereotypic[tw] OR gender stereotypical[tw] OR gender stereotypicality[tw] OR gender stereotyping[tw] OR gender bias[tw] OR gender issue[tw] OR gender issues[tw] OR gender factor[tw] OR gender factors[tw] OR gender variability[tw] OR gender variable[tw] OR gender variables[tw] OR gender variety[tw] OR gender varieties[tw] OR gender variation[tw] OR gender difference[tw] OR gender differences[tw] OR gender conflict[tw] OR gender conflicts[tw] OR gender diversity[tw] OR gender diversities[tw] OR gender analysis[tw] OR gender relation[tw] OR gender relational[tw] OR gender relations[tw] OR gender relationship[tw] OR gender relationships[tw] |
| #24 | Search sexism[tw] OR feminism[tw] OR sex factor[tw] OR sex factors[tw] |
| #23 | Search "sex factors"[MeSH Terms] OR "sexism"[MeSH Terms] OR "feminism"[MeSH Terms] |
| **#22** | **Search #19 OR #20 OR #21** |
| #21 | Search health care provider[tw] OR health care provider's[tw] OR health care providers[tw] OR doctor[tw] OR doctor's[tw] OR doctors[tw] OR doctors'[tw] OR practitioner[tw] OR practitioner's[tw] OR practitioners[tw] OR practitioners'[tw] OR physician[tw] OR physician's[tw] OR physicians[tw] OR physicians'[tw] OR clinician[tw] OR clinician's[tw] OR clinicians[tw] OR clinicians'[tw] OR staff[tw] OR specialist[tw] OR specialist's[tw] OR specialists[tw] OR specialists'[tw] OR surgeon[tw] OR surgeons[tw] OR surgeons'[tw] OR nurse[tw] OR nurse's[tw] OR nurses[tw] OR nurses'[tw] OR pharmacist[tw] OR pharmacist's[tw] OR pharmacists[tw] OR pharmacists'[tw] OR dentist[tw] OR dentist's[tw] OR dentistery[tw] OR dentists[tw] OR dentists'[tw] OR dietitian[tw] OR dietitian's[tw] OR dietitians[tw] OR dietitians'[tw] OR dietician[tw] OR dietician's[tw] OR dieticians[tw] OR dieticians'[tw] OR nutritionist[tw] OR nutritionist's[tw] OR nutritionists[tw] OR nutritionists'[tw] OR laboratory specialist[tw] OR laboratory specialistâ€™s[tw] OR laboratory specialists[tw] OR laboratory specialistsâ€™[tw] OR radiologist[tw] OR radiologist's[tw] OR radiologists[tw] OR radiologists'[tw] OR physical therapist[tw] OR physical therapists[tw] OR manager[tw] OR manager's[tw] OR managers[tw] OR managers'[tw] OR administrator[tw] OR administrator's[tw] OR administrators[tw] OR administrators'[tw] OR leader[tw] OR leader's[tw] OR leaders[tw] OR leadersâ€™[tw] OR leadership[tw] OR leadership's[tw] OR leaderships[tw] OR leaderships'[tw] OR leaderskills[tw] OR leaderstyle[tw] OR student[tw] OR student's[tw] OR students[tw] OR studentsâ€™[tw] OR trainee[tw] OR trainee's[tw] OR trainees[tw] OR trainees'[tw] OR intern[tw] OR intern's[tw] OR interns[tw] OR interns'[tw] |
| #20 | Search human capital[tw] OR human resource[tw] OR human resources[tw] OR worker[tw] OR worker's[tw] OR workers[tw] OR workers'[tw] OR workforce[tw] OR work-force[tw] OR manpower[tw] OR man-power[tw] OR personnel[tw] OR staff[tw] |
| #19 | Search "health manpower"[MeSH Terms] OR "health personnel"[MeSH Terms] OR "students, health occupations"[MeSH Terms] |
| **#18** | **Search #7 OR #8 OR #9 OR #10 OR #11 OR #12 OR #13 OR #14 OR #15 OR #16 OR #17** |
| #17 | Search need assessment[tw] OR need assessments[tw] OR needs assessment[tw] |
| #16 | Search "needs assessment"[MeSH Terms] |
| #15 | Search salaries[tw] OR salaries'[tw] OR salary[tw] OR salary's[tw] OR fringe[tw] OR incentive[tw] OR incentive's[tw] OR incentives[tw] OR incentives'[tw] OR income[tw] OR income's[tw] OR incomes[tw] OR wage[tw] OR wages[tw] OR wages'[tw] OR wages's[tw] OR pay equity[tw] OR sick leave[tw] OR sick leaves[tw] OR maternity leave[tw] OR maternity leaves[tw] OR maternal leave[tw] OR absenteeism[tw] OR absenteeisms[tw] |
| #14 | Search "income"[MeSH Terms:noexp] OR "salaries and fringe benefits"[MeSH Terms:noexp] OR "family leave"[MeSH Terms:noexp] OR "sick leave"[MeSH Terms] |
| #13 | Search turnover[tw] OR turn-over[tw] OR career mobility[tw] OR workload[tw] OR workload's[tw] OR workloadmanagement[tw] OR workloadpeak[tw] OR workloads[tw] |
| #12 | Search education[tw] OR learning[tw] OR preceptorship[tw] OR preceptorships[tw] OR preceptorships'[tw] OR mentorship[tw] OR mentorships[tw] OR residencies[tw] OR residencies'[tw] OR residency[tw] OR residency's[tw] OR residencyprograms[tw] OR internship[tw] OR internship's[tw] OR internships[tw] OR internships'[tw] OR training[tw] OR training's[tw] OR trainings[tw] OR trainings'[tw] OR orientation[tw] OR orientation's[tw] OR orientations[tw] OR orientations'[tw] OR development[tw] OR development's[tw] OR developmentalprograms[tw] OR developments[tw] OR developments'[tw] OR recruitment[tw] OR recruitment's[tw] OR recruitments[tw] OR retention[tw] OR retention's[tw] OR retentions[tw] OR retentions'[tw] OR retaining[tw] OR hiring[tw] OR planning[tw] OR management[tw] OR selection[tw] OR staffing[tw] OR scheduling[tw] OR motivation[tw] OR promotion[tw] OR performance[tw] |
| #11 | Search "personnel management"[MeSH Terms:noexp] OR "career mobility"[MeSH Terms] OR "employee incentive plans"[MeSH Terms] OR "personnel administration, hospital"[MeSH Terms] OR "personnel selection"[MeSH Terms] OR "personnel staffing and scheduling"[MeSH Terms] OR "personnel turnover"[MeSH Terms] OR "physician incentive plans"[MeSH Terms] OR "workload"[MeSH Terms] |
| #10 | Search "inservice training"[MeSH Terms] |
| #9 | Search "preceptorship"[MeSH Terms] |
| #8 | Search "learning"[MeSH Terms:noexp] |
| #7 | Search "education"[MeSH Terms:noexp] OR "curriculum"[MeSH Terms:noexp] OR "competency-based education"[MeSH Terms] OR "interdisciplinary studies"[MeSH Terms] OR "problem-based learning"[MeSH Terms] OR "education, predental"[MeSH Terms] OR "education, premedical"[MeSH Terms] OR "education, professional"[MeSH Terms:noexp] OR "education, continuing"[MeSH Terms] OR "education, dental"[MeSH Terms] OR "education, graduate"[MeSH Terms] OR "education, medical"[MeSH Terms] OR "education, nursing"[MeSH Terms] OR "education, pharmacy"[MeSH Terms] OR "education, public health professional"[MeSH Terms] OR "internship, nonmedical"[MeSH Terms] |
| **#5** | **Search #1 OR #2 OR #4** |
| #4 | Search medical center[tw] OR medical centers[tw] OR medical centre[tw] OR medical school[tw] OR medical schools[tw] OR medical facilities[tw] OR medical facility[tw] OR health center[tw] OR health centers[tw] OR health centre[tw] OR health school[tw] OR health schools[tw] OR health facilities[tw] OR health facility[tw] OR academic center[tw] OR academic centers[tw] OR academic centre[tw] OR academic school[tw] OR academic schools[tw] OR academic facilities[tw] OR academic facility[tw] OR hospital[tw] OR hospital's[tw] OR hospitals[tw] OR hospitalsâ€™[tw] |
| #2 | Search "health facilities"[MeSH Terms:noexp] OR "academic medical centers"[MeSH Terms] OR "health facility administration"[MeSH Terms] OR "hospital administration"[MeSH Terms] OR "hospital units"[MeSH Terms] OR "hospitals"[MeSH Terms] OR "laboratories, hospital"[MeSH Terms] OR "pharmacies"[MeSH Terms] |
| #1 | Search schools, health occupations[mesh] OR universities[mesh] |

| **Search** | **Query** |
| --- | --- |
| **All concepts combined** | **Search #5 AND #18 AND #22 AND #26**  Search **((schools, health occupations[mesh] OR universities[mesh]) OR ("health facilities"[MeSH Terms:noexp] OR "academic medical centers"[MeSH Terms] OR "health facility administration"[MeSH Terms] OR "hospital administration"[MeSH Terms] OR "hospital units"[MeSH Terms] OR "hospitals"[MeSH Terms] OR "laboratories, hospital"[MeSH Terms] OR "pharmacies"[MeSH Terms]) OR (medical center[tw] OR medical centers[tw] OR medical centre[tw] OR medical school[tw] OR medical schools[tw] OR medical facilities[tw] OR medical facility[tw] OR health center[tw] OR health centers[tw] OR health centre[tw] OR health school[tw] OR health schools[tw] OR health facilities[tw] OR health facility[tw] OR academic center[tw] OR academic centers[tw] OR academic centre[tw] OR academic school[tw] OR academic schools[tw] OR academic facilities[tw] OR academic facility[tw] OR hospital[tw] OR hospital's[tw] OR hospitals[tw] OR hospitals'[tw])) AND (("education"[MeSH Terms:noexp] OR "curriculum"[MeSH Terms:noexp] OR "competency-based education"[MeSH Terms] OR "interdisciplinary studies"[MeSH Terms] OR "problem-based learning"[MeSH Terms] OR "education, predental"[MeSH Terms] OR "education, premedical"[MeSH Terms] OR "education, professional"[MeSH Terms:noexp] OR "education, continuing"[MeSH Terms] OR "education, dental"[MeSH Terms] OR "education, graduate"[MeSH Terms] OR "education, medical"[MeSH Terms] OR "education, nursing"[MeSH Terms] OR "education, pharmacy"[MeSH Terms] OR "education, public health professional"[MeSH Terms] OR "internship, nonmedical"[MeSH Terms]) OR ("learning"[MeSH Terms:noexp]) OR ("preceptorship"[MeSH Terms]) OR ("inservice training"[MeSH Terms]) OR ("personnel management"[MeSH Terms:noexp] OR "career mobility"[MeSH Terms] OR "employee incentive plans"[MeSH Terms] OR "personnel administration, hospital"[MeSH Terms] OR "personnel selection"[MeSH Terms] OR "personnel staffing and scheduling"[MeSH Terms] OR "personnel turnover"[MeSH Terms] OR "physician incentive plans"[MeSH Terms] OR "workload"[MeSH Terms]) OR (education[tw] OR learning[tw] OR preceptorship[tw] OR preceptorships[tw] OR preceptorships'[tw] OR mentorship[tw] OR mentorships[tw] OR residencies[tw] OR residencies'[tw] OR residency[tw] OR residency's[tw] OR residencyprograms[tw] OR internship[tw] OR internship's[tw] OR internships[tw] OR internships'[tw] OR training[tw] OR training's[tw] OR trainings[tw] OR trainings'[tw] OR orientation[tw] OR orientation's[tw] OR orientations[tw] OR orientations'[tw] OR development[tw] OR development's[tw] OR developmentalprograms[tw] OR developments[tw] OR developments'[tw] OR recruitment[tw] OR recruitment's[tw] OR recruitments[tw] OR retention[tw] OR retention's[tw] OR retentions[tw] OR retentions'[tw] OR retaining[tw] OR hiring[tw] OR planning[tw] OR management[tw] OR selection[tw] OR staffing[tw] OR scheduling[tw] OR motivation[tw] OR promotion[tw] OR performance[tw]) OR (turnover[tw] OR turn-over[tw] OR career mobility[tw] OR workload[tw] OR workload's[tw] OR workloadmanagement[tw] OR workloadpeak[tw] OR workloads[tw]) OR ("income"[MeSH Terms:noexp] OR "salaries and fringe benefits"[MeSH Terms:noexp] OR "family leave"[MeSH Terms:noexp] OR "sick leave"[MeSH Terms]) OR (salaries[tw] OR salaries'[tw] OR salary[tw] OR salary's[tw] OR fringe[tw] OR incentive[tw] OR incentive's[tw] OR incentives[tw] OR incentives'[tw] OR income[tw] OR income's[tw] OR incomes[tw] OR wage[tw] OR wages[tw] OR wages'[tw] OR wages's[tw] OR pay equity[tw] OR sick leave[tw] OR sick leaves[tw] OR maternity leave[tw] OR maternity leaves[tw] OR maternal leave[tw] OR absenteeism[tw] OR absenteeisms[tw]) OR ("needs assessment"[MeSH Terms]) OR (need assessment[tw] OR need assessments[tw] OR needs assessment[tw])) AND (("health manpower"[MeSH Terms] OR "health personnel"[MeSH Terms] OR "students, health occupations"[MeSH Terms]) OR (human capital[tw] OR human resource[tw] OR human resources[tw] OR worker[tw] OR worker's[tw] OR workers[tw] OR workers'[tw] OR workforce[tw] OR work-force[tw] OR manpower[tw] OR man-power[tw] OR personnel[tw] OR staff[tw]) OR (health care provider[tw] OR health care provider's[tw] OR health care providers[tw] OR doctor[tw] OR doctor's[tw] OR doctors[tw] OR doctors'[tw] OR practitioner[tw] OR practitioner's[tw] OR practitioners[tw] OR practitioners'[tw] OR physician[tw] OR physician's[tw] OR physicians[tw] OR physicians'[tw] OR clinician[tw] OR clinician's[tw] OR clinicians[tw] OR clinicians'[tw] OR staff[tw] OR specialist[tw] OR specialist's[tw] OR specialists[tw] OR specialists'[tw] OR surgeon[tw] OR surgeons[tw] OR surgeons'[tw] OR nurse[tw] OR nurse's[tw] OR nurses[tw] OR nurses'[tw] OR pharmacist[tw] OR pharmacist's[tw] OR pharmacists[tw] OR pharmacists'[tw] OR dentist[tw] OR dentist's[tw] OR dentistery[tw] OR dentists[tw] OR dentists'[tw] OR dietitian[tw] OR dietitian's[tw] OR dietitians[tw] OR dietitians'[tw] OR dietician[tw] OR dietician's[tw] OR dieticians[tw] OR dieticians'[tw] OR nutritionist[tw] OR nutritionist's[tw] OR nutritionists[tw] OR nutritionists'[tw] OR laboratory specialist[tw] OR laboratory specialist's[tw] OR laboratory specialists[tw] OR laboratory specialists'[tw] OR radiologist[tw] OR radiologist's[tw] OR radiologists[tw] OR radiologists'[tw] OR physical therapist[tw] OR physical therapists[tw] OR manager[tw] OR manager's[tw] OR managers[tw] OR managers'[tw] OR administrator[tw] OR administrator's[tw] OR administrators[tw] OR administrators'[tw] OR leader[tw] OR leader's[tw] OR leaders[tw] OR leaders'[tw] OR leadership[tw] OR leadership's[tw] OR leaderships[tw] OR leaderships'[tw] OR leaderskills[tw] OR leaderstyle[tw] OR student[tw] OR student's[tw] OR students[tw] OR students'[tw] OR trainee[tw] OR trainee's[tw] OR trainees[tw] OR trainees'[tw] OR intern[tw] OR intern's[tw] OR interns[tw] OR interns'[tw])) AND (("sex factors"[MeSH Terms] OR "sexism"[MeSH Terms] OR "feminism"[MeSH Terms]) OR (sexism[tw] OR feminism[tw] OR sex factor[tw] OR sex factors[tw]) OR (gender sensitivity[tw] OR gender sensitive[tw] OR gender equitable[tw] OR gender equity[tw] OR gender inequitable[tw] OR gender inequities[tw] OR gender inequity[tw] OR gender equalitarian[tw] OR gender equality[tw] OR gender inequalities[tw] OR gender inequality[tw] OR gender budgeting[tw] OR gender mainstreaming[tw] OR gender discriminated[tw] OR gender discriminating[tw] OR gender discrimination[tw] OR gender discriminations[tw] OR gender discriminative[tw] OR gender discriminatory[tw] OR gender stereotype[tw] OR gender stereotyped[tw] OR gender stereotypes[tw] OR gender stereotypic[tw] OR gender stereotypical[tw] OR gender stereotypicality[tw] OR gender stereotyping[tw] OR gender bias[tw] OR gender issue[tw] OR gender issues[tw] OR gender factor[tw] OR gender factors[tw] OR gender variability[tw] OR gender variable[tw] OR gender variables[tw] OR gender variety[tw] OR gender varieties[tw] OR gender variation[tw] OR gender difference[tw] OR gender differences[tw] OR gender conflict[tw] OR gender conflicts[tw] OR gender diversity[tw] OR gender diversities[tw] OR gender analysis[tw] OR gender relation[tw] OR gender relational[tw] OR gender relations[tw] OR gender relationship[tw] OR gender relationships[tw]))** |

# Scopus

Scopus Exact Search Strategy:

[( ( TITLE-ABS-KEY ( **sexism**  OR  **feminism** ) )  OR  ( TITLE-ABS-KEY ( ( **gender**  OR  **sex** )  PRE/1  ( **sensitivity** OR  **equity OR equities**  OR  **inequity**  OR inequities OR  **equality**  OR equalities OR  **inequality OR inequalities**  OR   **mainstreaming**  OR **discrimination**  OR  **bias** OR **difference**  OR **conflict**) ) ) )  AND  ( ( TITLE-ABS-KEY ( **curriculum**  OR **education**  OR  **learning**  OR  **preceptorship**  OR  **mentorship**  OR  **residency**   OR  **internship** OR  **training**  OR  **orientation**  OR  **development**  OR  **recruitment**  OR  **retention**  OR  **retaining** OR  **hiring**  OR  **planning**  OR  **management**  OR  **selection**  OR  **staffing**  OR  **scheduling**) )  OR  ( TITLE-ABS-KEY ( **turnover**  OR  **workload** ) )  OR  ( TITLE-ABS-KEY (**career**  PRE/1  **mobility** ) )  OR  ( TITLE-ABS-KEY ( **salary**  OR  **fringe**  OR **incentive**  OR  **income**  OR  **wage**  OR  **absenteeism** ) )  OR  ( TITLE-ABS-KEY ( ( **pay**  PRE/1  **equit*** )  OR ( **sick**  PRE/1  **leave** )  OR  ( **maternity**  pre/1  **leave** ) ) )  OR  ( TITLE-ABS-KEY ( **need**  PRE/1  **assessment** ) ) ) AND  ( ( TITLE-ABS-KEY ( ( **medical**  OR  **health**  OR  **academic** )  PRE/2  ( **university**  OR  **center**  OR **centre**  OR  **school**  OR  **facility** ) ) )  OR  ( TITLE-ABS-KEY ( **hospital** ) ) )  AND  ( ( TITLE-ABS-KEY ( **human** PRE/2  ( **capital**  OR  **resource** ) ) )  OR  ( TITLE-ABS-KEY ( **worker**  OR  **workforce**  OR **manpower**  OR **personnel**  OR  **staff** ) )  OR  ( TITLE-ABS-KEY ( ( **work**  PRE/1  **force** )  OR  ( **man**  PRE/1  **power** ) ) )  OR ( TITLE-ABS-KEY ( ( **healthcare**  PRE/1  **provider** )  OR  ( **medical**  PRE/1  **lab**  PRE/1 **specialist** )  OR  ( **physical**  PRE/1  **therapist** ) ) )  OR  ( TITLE-ABS-KEY ( **doctor**  OR  **practitioner**  OR **physician**  OR  **clinician**  OR  **staff**  OR  **specialist**  OR  **surgeon**  OR  **nurse**  OR  **pharmacist**  OR **dentist**  OR  **dieti?ian**  OR  **nutritionist**  OR  **radiologist**  OR  **manager**  OR  **administrator**  OR **leader**  OR  **student**  OR  **trainee**  OR  **intern** ) ) )](http://www.scopus.com.ezproxy.aub.edu.lb/search/save/action.uri?activity=allAction&userSearchID=5&origin=savedsearch)

***Scopus Search Strategy divided by concepts:***

**CONCEPT 1:**

( ( TITLE-ABS-KEY ( sexism  OR  feminism ) )  OR  ( TITLE-ABS-KEY ( ( gender  OR  sex )  PRE/1  ( sensitivity OR  equity OR equities  OR  inequity  OR inequities OR  equality  OR equalities OR  inequality OR inequalities  OR   mainstreaming  OR discrimination  OR  bias OR difference  OR conflict) ) ) )

**CONCEPT 2:**

( ( TITLE-ABS-KEY ( curriculum  OR education  OR  learning  OR  preceptorship  OR  mentorship  OR  residency   OR  internship OR  training  OR  orientation  OR  development  OR  recruitment  OR  retention  OR  retaining OR  hiring  OR  planning  OR  management  OR  selection  OR  staffing  OR  scheduling) )  OR  ( TITLE-ABS-KEY ( turnover  OR  workload ) )  OR  ( TITLE-ABS-KEY (career  PRE/1  mobility ) )  OR  ( TITLE-ABS-KEY ( salary  OR  fringe  OR incentive  OR  income  OR  wage  OR  absenteeism ) )  OR  ( TITLE-ABS-KEY ( ( pay  PRE/1  equit* )  OR ( sick  PRE/1  leave )  OR  ( maternity  pre/1  leave ) ) )  OR  ( TITLE-ABS-KEY ( need  PRE/1  assessment ) ) )

--------------------------------------------------------------------------------

**CONCEPT 3:**

( ( TITLE-ABS-KEY ( ( medical  OR  health  OR  academic )  PRE/2  ( university  OR  center  OR centre  OR  school  OR  facility ) ) )  OR  ( TITLE-ABS-KEY ( hospital ) ) )

--------------------------------------------------------------------------------

**CONCEPT 4:**

( ( TITLE-ABS-KEY ( human PRE/2  ( capital  OR  resource ) ) )  OR  ( TITLE-ABS-KEY ( worker  OR  workforce  OR manpower  OR personnel  OR  staff ) )  OR  ( TITLE-ABS-KEY ( ( work  PRE/1  force )  OR  ( man  PRE/1  power ) ) )  OR ( TITLE-ABS-KEY ( ( healthcare  PRE/1  provider )  OR  ( medical  PRE/1  lab  PRE/1 specialist )  OR  ( physical  PRE/1  therapist ) ) )  OR  ( TITLE-ABS-KEY ( doctor  OR  practitioner  OR physician  OR  clinician  OR  staff  OR  specialist  OR  surgeon  OR  nurse  OR  pharmacist  OR dentist  OR  dieti?ian  OR  nutritionist  OR  radiologist  OR  manager  OR  administrator  OR leader  OR  student  OR  trainee  OR  intern ) ) )

***************************

# CINAHL

| Search ID | Search Terms |
| --- | --- |
| S31 | S7 AND S23 AND S24 AND S30 |
| S30 | S25 OR S26 OR S27 OR S28 OR S29 |
| S29 | TI (health W1 care W1 provider*) OR TI (healthcare W1 provider*) OR TI  doctor* OR TI practitioner* OR TI physician* OR TI clinician* OR TI staff OR TI specialist* OR TI surgeon* OR TI nurse* OR TI pharmacist* OR  TI dentist* OR TI dietitian* OR TI dietician* OR TI nutritionist* OR TI (laboratory W1 specialist*) OR TI radiologist* OR TI (physical W1  therapist*) OR TI manager* OR TI administrator* OR TI leader* OR TI student* OR TI trainee* OR TI intern* OR AB (health W1 care W1  provider*) OR AB (h ... |
| S28 | TI worker* OR TI (work W1 orce) OR TI workforce OR TI (man W1 power) OR TI manpower OR TI personnel OR TI staff OR AB worker* OR AB (work W1 force) OR AB workforce OR AB (man W1 power) OR AB manpower OR AB personnel OR AB staff |
| S27 | TI human W2 (TI capital OR TI resource*) OR AB human W2 (AB capital OR AB resource*) |
| S26 | (MH "Students, Nursing, Graduate") OR (MH "Students, Health Occupations+") OR (MH "Students, Nursing+") OR (MH "Students, Nursing, Male") OR (MH "Students, Nursing, Practical") OR (MH Students, Pharmacy") OR (MH "Students, Podiatry") OR (MH "Students, Pre-Nursing") |
| S25 | (MH "Health Manpower") OR (MH "Allied Health Personnel") OR (MH "Administrative Personnel") OR (MH "Alternative Health Personnel") OR (MH "Community Health Workers") OR (MH "Consultants") OR (MH "Lactation Consultants") OR (MH "Nurse Consultants") OR (MH "Foreign  Professional Personnel+") OR (MH "Health Personnel") OR (MH "Coroners and Medical Examiners") OR (MH "Dentists") OR (MH "Faculty, Dental") OR (MH "Faculty, Medical") OR (MH "Faculty, Nursing") OR (MH "Health Facility Administrators") OR … |
| S24 | S19 OR S20 OR S21 OR S22 |
| S23 | S8 OR S9 OR S10 OR S11 OR S12 OR S13 OR S14 OR S15 OR S16 OR S17 OR S18 |
| S22 | (AB medical OR AB health OR AB academic) W2 (AB center* OR AB centre OR AB school* OR AB facilit*) OR AB hospital* |
| S21 | (TI medical OR TI health OR TI academic) W2 (TI center* OR TI centre OR TI school* OR TI facilit*) OR TI hospital* |
| S20 | (MH "Schools, Dental") OR (MH "Schools, Medical") OR (MH "Schools, Nursing") OR (MH "Schools, Podiatry") OR (MH "Schools, Allied Health") OR (MH "Health Facilities") OR (MH "Academic Medical Centers") OR (MH "Alternative Health Facilities") OR (MH "Dental Facilities") OR (MH "Nursing Service") OR (MH "Obstetric Service") OR (MH "Occupational  Therapy Service") OR (MH "Outpatient Service") OR (MH "Pharmacy Service") OR (MH "Radiology Service") OR (MH "Physical Therapy Service") OR (MH "Respiratory ... |
| S19 | (MH "Schools, Health Occupations+") |
| S18 | TI (need* N2 assessment*) OR AB (need* N2 assessment*) |
| S17 | TI salar* OR TI fringe* OR TI incentive* OR TI income* OR TI wage* OR TI (pay N2 equit*) OR TI (sick W1 leave*) OR TI (matern* W1 leave*) OR TI absenteeism OR AB salar* OR AB fringe* OR AB incentive* OR AB income* OR AB wage* OR AB (pay N2 equit*) OR AB (sick W1 leave*) OR AB (matern* W1 leave*) OR AB absenteeism |
| S16 | TI turnover OR TI (turn W1 over) OR TI (career W1 mobility) OR TI workload* OR TI (work N2 load*) OR AB turnover OR AB (turn W1 over) OR AB (career W1 mobility) OR AB workload* OR AB (work N2 load*) |
| S15 | TI education OR TI learning OR TI preceptorship* OR TI mentorship* OR TI residenc* OR TI internship* OR TI training* OR TI orientation* OR TI development* OR TI recruitment* OR TI retention* OR TI retaining OR TI hiring OR TI planning OR TI management OR TI selection OR TI staffing R TI scheduling OR TI motivation OR TI promotion OR TI performance OR AB education OR AB learning OR AB preceptorship* OR AB mentorship* OR AB residenc* OR AB internship* OR AB training* OR AB orientation* OR AB deve ... |
| S14 | (MH "Needs Assessment") |
| S13 | (MH "Income") OR (MH "Salaries and Fringe Benefits") OR (MH "Family and Medical Leave+") OR (MH "Health Benefit Plans, Employee") OR (MH "Pensions") |
| S12 | (MH "Personnel Management") OR (MH "Health Services Administration")  OR (MH "Health Facility Administration+") OR (MH "Nursing  Administration+") OR (MH "Career Mobility") OR (MH "Employee Incentive  Programs") OR (MH "Personnel Selection") OR (MH "Personnel Staffing and Scheduling") OR (MH "Personnel Turnover") OR (MH "Physician Incentive Plans") OR (MH "Salaries and Fringe Benefits") OR (MH "Family  and Medical Leave+") OR (MH "Health Benefit Plans, Employee") OR (MH "Staff Development") OR (MH " ... |
| S11 | (MH "Staff Development") |
| S10 | (MH "Learning") |
| S9 | (MH "Curriculum") OR (MH "Education") OR (MH "Education, Clinical") OR (MH "Education, Competency- Based") OR (MH "Nutrition Education") OR (MH "Dental Health Education") OR (MH "Health Education") OR (MH "Childbirth Education") OR (MH "Education, Health Sciences") OR (MH "Education, Continuing+") OR (MH "Education, Dental") OR (MH "Education, Graduate+") OR (MH "Education, Interdisciplinary") OR (MH  "Education, Medical+") OR (MH "Education, Midwifery") OR (MH Education, Nursing+") OR (MH "Educa ... |
| S8 | (MH "Education+") |
| S7 | (S1 OR S2 OR S3 OR S4 OR S5 OR S6) |
| S6 | TI feminism OR AB feminism |
| S5 | TI sexism OR AB sexism |
| S4 | (TI gender OR AB gender OR TI sex OR AB sex) W2 (TI sensitiv* OR TI  equit* OR TI inequit* OR TI equalit* OR TI inequalit* OR TI budget* OR TI  mainstream* OR TI discriminat* OR TI stereotyp* OR TI bias OR TI issue* OR TI factor* OR TI variab* OR TI difference* OR TI conflict* OR TI diversit* OR TI analysis OR TI relation* OR AB sensitiv* OR AB equit* OR AB inequit* OR AB equalit* OR AB inequalit* OR AB budget* OR AB mainstream* OR AB discriminat* OR AB stereotyp* OR AB bias OR AB  issue* OR AB fac ... |
| S3 | (MH "Feminism+") |
| S2 | (MH "Sexism+") OR (MH "Gender Bias") |
| S1 | (MH "Sex Factors") OR (MH "Gender Identity+") |

# Sociological Abstracts:

**Concept 1**

(SU.EXACT("Sex Stereotypes") OR SU.EXACT("Sex Differences") OR SU.EXACT("Sexism") OR SU.EXACT("Occupational Segregation") OR SU.EXACT("Sexual Inequality") OR SU.EXACT("Feminism")) OR (AB,TI("sex stereotyp*" OR "sex differenc*" OR sexism OR "occupational segregation" OR "sexual inequalit*" OR feminism)) OR (AB,TI((gender OR sex) PRE/2 (sensitiv* OR equit* OR inequit* OR equalit* OR inequalit* OR budget* OR mainstream* OR discriminat* OR stereotyp* OR bias OR issue* OR factor* OR variab* OR difference* OR conflict* OR diversit* OR analysis OR relation*)))

**Concept 2**

(SU.EXACT("Occupational Choice") OR SU.EXACT("Job Training") OR SU.EXACT("Educational Programs") OR SU.EXACT("Wages") OR SU.EXACT("Higher Education") OR SU.EXACT("Vocational Education") OR SU.EXACT("Employment Discrimination") OR SU.EXACT("Masters Programs") OR SU.EXACT("Budgets") OR SU.EXACT("Income Inequality") OR SU.EXACT("Professional Consultation") OR SU.EXACT("Promotion (Occupational)") OR SU.EXACT("Income") OR SU.EXACT("Adult Education") OR SU.EXACT("Curriculum") OR SU.EXACT("Fellowships and Scholarships") OR SU.EXACT("Occupational Mobility") OR SU.EXACT("Encouragement") OR SU.EXACT("Graduate Students") OR SU.EXACT("Education") OR SU.EXACT("Personnel Management") OR SU.EXACT("Professional Training") OR SU.EXACT("Employment") OR SU.EXACT("Recruitment") OR SU.EXACT("Doctoral Programs") OR SU.EXACT("Guidance") OR SU.EXACT("Motivation") OR SU.EXACT("Pensions") OR SU.EXACT("Incentives") OR SU.EXACT("Education Work Relationship") OR SU.EXACT("Work Orientations") OR SU.EXACT("Salaries") OR SU.EXACT("Training") OR SU.EXACT("Income Distribution") OR SU.EXACT("Employment Opportunities") OR SU.EXACT("Rewards")) OR (AB,TI("Occupation* Choic*" OR "Job Training" OR "Educational Program*" OR "Wage*" OR "Higher Education" OR "Vocational Education" OR "Employment Discriminat* " OR " Masters Program* " OR "Budget*" OR "Income Inequal*" OR "Professional Consultation" OR "Promotion*" OR "Income" OR "Adult Education" OR "Curriculum" OR "Fellowship*" OR "Scholarship*" OR "Occupation* Mobility" OR "Encourag*" OR "Education" OR "Personnel Management" OR "Professional Training" OR "Employment" OR "Recruitment" OR "Doctoral Program*" OR "Guidance" OR "Motivation" OR "Pension*" OR "Incentiv*" OR "Education Work Relationship" OR "Work Orientation*" OR "Salar*" OR "Training" OR "Income Distribution" OR "Employment Opportunit*" OR "Reward*")) OR (AB,TI(education OR learning OR preceptorship* OR mentorship* OR residenc* OR internship* OR training* OR orientation* OR development OR recruitment* OR retention* OR retaining OR hiring OR planning OR management OR selection OR staffing OR scheduling OR motivation OR promotion OR performance)) OR (AB,TI(turnover OR "turn PRE/0 over" OR "turn over" OR "career PRE/0 mobility" OR "career mobility" OR workload* OR "work PRE/0 load*" OR "work load")) OR (AB,TI(salar* OR fringe* OR incentive* OR income* OR wage* OR "pay PRE/1 equit*" OR ((sick OR matern*) PRE/1 leave*)))

**Concept 3**

(SU.EXACT("Universities") OR SU.EXACT("Hospices") OR SU.EXACT("Laboratories") OR SU.EXACT("Medical Schools") OR SU.EXACT("Hospitals") OR SU.EXACT("Nursing Homes") OR SU.EXACT("Professional Training")) OR (AB,TI(universit* OR hospices OR laborator* OR hospital*) OR AB,TI("nursing homes" OR "nursing home" OR "medical school" OR "medical schools")) OR AB,TI("Health PRE/2 facilities" OR "health PRE/2 facility" OR "medical PRE/2 center" OR "medical PRE/2 centers" OR "medical PRE/2 centre" OR "medical PRE/2 centres" OR "medical PRE/2 facility" OR "medical PRE/2 facilities" OR pharmac* OR "health PRE/2 center" OR "health PRE/2 centers" OR "health PRE/2 centre" OR "health PRE/2 centres" OR "health PRE/2 facility" OR "health PRE/2 facilities" OR "medical PRE/2 hospital" OR "care PRE/1 centers" OR "care PRE/1 center" OR "care PRE/1 centre" OR "care PRE/1 centres")

**Concept 4**

(SU.EXACT("Therapists") OR SU.EXACT("Paramedical Personnel") OR SU.EXACT("Management") OR SU.EXACT("Workers") OR SU.EXACT("Psychologists") OR SU.EXACT("Policy Making") OR SU.EXACT("Medical Students") OR SU.EXACT("Dentists") OR SU.EXACT("Pharmacists") OR SU.EXACT("Managers") OR SU.EXACT("Caregivers") OR SU.EXACT("Psychiatrists") OR SU.EXACT("Nurses") OR SU.EXACT("Labor Force") OR SU.EXACT("Human Capital") OR SU.EXACT("Physicians") OR SU.EXACT("Human Resources") OR SU.EXACT("Health Professions") OR SU.EXACT("Administrators" OR "Deans" OR "Directors" OR "Executives" OR "Managers" OR "Principals" OR "Superintendents")) OR (AB,TI(Therapist* OR Worker* OR staff OR Psychologist* OR Dentist* OR Pharmacist* OR Manager* OR Caregiver* OR Psychiatrist* OR Nurses OR Nurse Physician* OR Administrator* OR Dean* OR Director* OR Executive* OR Manager*) OR AB,TI("Paramedical Personnel")) OR (AB,TI("policy makers") OR AB,TI("policy maker")) OR (AB,TI("medical student") OR AB,TI("medical students")) OR (AB,TI("labor force") OR AB,TI("human capital")) OR (AB,TI("human resources") OR AB,TI("Health professional")) OR (AB,TI("health professionals") OR AB,TI("health professions")) OR (AB,TI("health PRE/1 care PRE/1 provider") OR ((health adj care adj provider*) or doctor* or practitioner* or physician* or clinician* or staff or specialist* or surgeon* or nurse* or pharmacist* or dentist* or dieti?ian* or nutritionist* or (medical adj lab adj specialist*) or radiologist* or physical therapist* or manager* or administrator* or leader* or student* or trainee* or intern*).) OR (AB,TI("medical PRE/1 lab PRE/1 specialist" ) OR AB,TI(physical therapists)) OR (AB,TI("physical therapist") OR AB,TI(Practitioners or clinician or clinicians or staff or specialists or specialist or surgeon or surgeons or dietician or dieticians or dietitian or dietitians or doctor or doctors or radiologist or radiologists or practitioner or manager or managers or administrator or administrators or leader or leaders or student or students or trainee or trainees or intern or fellow))

**Concept 5**

((SU.EXACT("Therapists") OR SU.EXACT("Paramedical Personnel") OR SU.EXACT("Management") OR SU.EXACT("Workers") OR SU.EXACT("Psychologists") OR SU.EXACT("Policy Making") OR SU.EXACT("Medical Students") OR SU.EXACT("Dentists") OR SU.EXACT("Pharmacists") OR SU.EXACT("Managers") OR SU.EXACT("Caregivers") OR SU.EXACT("Psychiatrists") OR SU.EXACT("Nurses") OR SU.EXACT("Labor Force") OR SU.EXACT("Human Capital") OR SU.EXACT("Physicians") OR SU.EXACT("Human Resources") OR SU.EXACT("Health Professions") OR SU.EXACT("Administrators" OR "Deans" OR "Directors" OR "Executives" OR "Managers" OR "Principals" OR "Superintendents")) OR (AB,TI(Therapist* OR Worker* OR staff OR Psychologist* OR Dentist* OR Pharmacist* OR Manager* OR Caregiver* OR Psychiatrist* OR Nurses OR Nurse Physician* OR Administrator* OR Dean* OR Director* OR Executive* OR Manager*) OR AB,TI("Paramedical Personnel")) OR (AB,TI("policy makers") OR AB,TI("policy maker")) OR (AB,TI("medical student") OR AB,TI("medical students")) OR (AB,TI("labor force") OR AB,TI("human capital")) OR (AB,TI("human resources") OR AB,TI("Health professional")) OR (AB,TI("health professionals") OR AB,TI("health professions")) OR (AB,TI("health PRE/1 care PRE/1 provider") OR ((health adj care adj provider*) OR doctor* OR practitioner* OR physician* OR clinician* OR staff OR specialist* OR surgeon* OR nurse* OR pharmacist* OR dentist* OR dieti?ian* OR nutritionist* OR (medical adj lab adj specialist*) OR radiologist* OR physical therapist* OR manager* OR administrator* OR leader* OR student* OR trainee* OR intern*) .) OR (AB,TI("medical PRE/1 lab PRE/1 specialist") OR AB,TI(physical therapists)) OR (AB,TI("physical therapist") OR AB,TI(Practitioners OR clinician OR clinicians OR staff OR specialists OR specialist OR surgeon OR surgeons OR dietician OR dieticians OR dietitian OR dietitians OR doctor OR doctors OR radiologist OR radiologists OR practitioner OR manager OR managers OR administrator OR administrators OR leader OR leaders OR student OR students OR trainee OR trainees OR intern OR fellow))) AND ((SU.EXACT("Universities") OR SU.EXACT("Hospices") OR SU.EXACT("Laboratories") OR SU.EXACT("Medical Schools") OR SU.EXACT("Hospitals") OR SU.EXACT("Nursing Homes") OR SU.EXACT("Professional Training")) OR (AB,TI(universit* OR hospices OR laborator* OR hospital*) OR AB,TI("nursing homes" OR "nursing home" OR "medical school" OR "medical schools")) OR AB,TI("Health PRE/2 facilities" OR "health PRE/2 facility" OR "medical PRE/2 center" OR "medical PRE/2 centers" OR "medical PRE/2 centre" OR "medical PRE/2 centres" OR "medical PRE/2 facility" OR "medical PRE/2 facilities" OR pharmac* OR "health PRE/2 center" OR "health PRE/2 centers" OR "health PRE/2 centre" OR "health PRE/2 centres" OR "health PRE/2 facility" OR "health PRE/2 facilities" OR "medical PRE/2 hospital" OR "care PRE/1 centers" OR "care PRE/1 center" OR "care PRE/1 centre" OR "care PRE/1 centres")) AND ((SU.EXACT("Occupational Choice") OR SU.EXACT("Job Training") OR SU.EXACT("Educational Programs") OR SU.EXACT("Wages") OR SU.EXACT("Higher Education") OR SU.EXACT("Vocational Education") OR SU.EXACT("Employment Discrimination") OR SU.EXACT("Masters Programs") OR SU.EXACT("Budgets") OR SU.EXACT("Income Inequality") OR SU.EXACT("Professional Consultation") OR SU.EXACT("Promotion (Occupational)") OR SU.EXACT("Income") OR SU.EXACT("Adult Education") OR SU.EXACT("Curriculum") OR SU.EXACT("Fellowships and Scholarships") OR SU.EXACT("Occupational Mobility") OR SU.EXACT("Encouragement") OR SU.EXACT("Graduate Students") OR SU.EXACT("Education") OR SU.EXACT("Personnel Management") OR SU.EXACT("Professional Training") OR SU.EXACT("Employment") OR SU.EXACT("Recruitment") OR SU.EXACT("Doctoral Programs") OR SU.EXACT("Guidance") OR SU.EXACT("Motivation") OR SU.EXACT("Pensions") OR SU.EXACT("Incentives") OR SU.EXACT("Education Work Relationship") OR SU.EXACT("Work Orientations") OR SU.EXACT("Salaries") OR SU.EXACT("Training") OR SU.EXACT("Income Distribution") OR SU.EXACT("Employment Opportunities") OR SU.EXACT("Rewards")) OR (AB,TI("Occupation* Choic*" OR "Job Training" OR "Educational Program*" OR "Wage*" OR "Higher Education" OR "Vocational Education" OR "Employment Discriminat* " OR " Masters Program* " OR "Budget*" OR "Income Inequal*" OR "Professional Consultation" OR "Promotion*" OR "Income" OR "Adult Education" OR "Curriculum" OR "Fellowship*" OR "Scholarship*" OR "Occupation* Mobility" OR "Encourag*" OR "Education" OR "Personnel Management" OR "Professional Training" OR "Employment" OR "Recruitment" OR "Doctoral Program*" OR "Guidance" OR "Motivation" OR "Pension*" OR "Incentiv*" OR "Education Work Relationship" OR "Work Orientation*" OR "Salar*" OR "Training" OR "Income Distribution" OR "Employment Opportunit*" OR "Reward*")) OR (AB,TI(education OR learning OR preceptorship* OR mentorship* OR residenc* OR internship* OR training* OR orientation* OR development OR recruitment* OR retention* OR retaining OR hiring OR planning OR management OR selection OR staffing OR scheduling OR motivation OR promotion OR performance)) OR (AB,TI(turnover OR "turn PRE/0 over" OR "turn over" OR "career PRE/0 mobility" OR "career mobility" OR workload* OR "work PRE/0 load*" OR "work load")) OR (AB,TI(salar* OR fringe* OR incentive* OR income* OR wage* OR "pay PRE/1 equit*" OR ((sick OR matern*) PRE/1 leave*)))) AND ((SU.EXACT("Sex Stereotypes") OR SU.EXACT("Sex Differences") OR SU.EXACT("Sexism") OR SU.EXACT("Occupational Segregation") OR SU.EXACT("Sexual Inequality") OR SU.EXACT("Feminism")) OR (AB,TI("sex stereotyp*" OR "sex differenc*" OR sexism OR "occupational segregation" OR "sexual inequalit*" OR feminism)) OR (AB,TI((gender OR sex) PRE/2 (sensitiv* OR equit* OR inequit* OR equalit* OR inequalit* OR budget* OR mainstream* OR discriminat* OR stereotyp* OR bias OR issue* OR factor* OR variab* OR difference* OR conflict* OR diversit* OR analysis OR relation*)))) AND pd(>19960101)

# Cochrane

Search Name: Gender Sensitivity & HR - All concepts + ANDing

Description:

ID Search

#1 MeSH descriptor: [Sex Factors] explode all trees

#2 MeSH descriptor: [Sexism] explode all trees

#3 MeSH descriptor: [Feminism] explode all trees

#4 sexism:ti,ab,kw (Word variations have been searched)

#5 feminism:ti,ab,kw (Word variations have been searched)

#6 "Sex factors":ti,ab,kw (Word variations have been searched)

#7 (gender or sex) near/2 (sensitiv* or equit* or inequit* or equalit* or inequalit* or budget* or mainstream* or discriminat* or stereotyp* or bias or issue* or factor* or variab* or difference* or conflict* or diversit* or analysis or relation*):ti,ab,kw (Word variations have been searched)

#8 {or #1-#7}

#9 MeSH descriptor: [Education] explode all trees

#10 MeSH descriptor: [Curriculum] explode all trees

#11 MeSH descriptor: [Learning] explode all trees

#12 MeSH descriptor: [Preceptorship] explode all trees

#13 MeSH descriptor: [Inservice Training] explode all trees

#14 MeSH descriptor: [Staff Development] explode all trees

#15 MeSH descriptor: [Personnel Management] explode all trees

#16 MeSH descriptor: [Career Mobility] explode all trees

#17 MeSH descriptor: [Salaries and Fringe Benefits] explode all trees

#18 MeSH descriptor: [Needs Assessment] explode all trees

#19 MeSH descriptor: [Income] explode all trees

#20 education or learning or preceptorship* or mentorship* or residenc* or internship* or training* or orientation* or development or recruitment* or retention* or retaining or hiring or planning or management or selection or staffing or scheduling or motivation or promotion or performance:ti,ab,kw (Word variations have been searched)

#21 turnover or "turn over" or "career mobility" or workload or "work load":ti,ab,kw (Word variations have been searched)

#22 turn near/1 over:ti,ab,kw (Word variations have been searched)

#23 career near/1 mobility:ti,ab,kw (Word variations have been searched)

#24 work near/1 load:ti,ab,kw (Word variations have been searched)

#25 pay near/1 equit*:ti,ab,kw (Word variations have been searched)

#26 (Sick or maternal) near/1 leave*:ti,ab,kw (Word variations have been searched)

#27 salar* or fringe* or incentive* or income* or wage* or absenteeism:ti,ab,kw (Word variations have been searched)

#28 "Needs assessment":ti,ab,kw

#29 {or #9-#28}

#30 MeSH descriptor: [Schools, Health Occupations] explode all trees

#31 MeSH descriptor: [Health Facilities] explode all trees

#32 MeSH descriptor: [Academic Medical Centers] explode all trees

#33 MeSH descriptor: [Hospitals] explode all trees

#34 MeSH descriptor: [Laboratories] explode all trees

#35 MeSH descriptor: [Hospital Units] explode all trees

#36 MeSH descriptor: [Hospital Departments] explode all trees

#37 (medical or health or academic) near/2 (center* or centre or school* or facilit*):ti,ab,kw (Word variations have been searched)

#38 laborator* or hospital*:ti,ab,kw

#39 {or #30-#38}

#40 MeSH descriptor: [Health Manpower] explode all trees

#41 MeSH descriptor: [Health Personnel] explode all trees

#42 MeSH descriptor: [Physicians] explode all trees

#43 MeSH descriptor: [Nurses] explode all trees

#44 MeSH descriptor: [Students, Health Occupations] explode all trees

#45 human near/2 (capital or resource*):ti,ab,kw (Word variations have been searched)

#46 worker* or workforce or manpower or personnel or staff:ti,ab,kw (Word variations have been searched)

#47 work near/1 force:ti,ab,kw (Word variations have been searched)

#48 man near/1 power:ti,ab,kw (Word variations have been searched)

#49 doctor* or practitioner* or physician* or clinician* or staff or specialist* or surgeon* or nurse* or pharmacist* or dentist* or dieti?ian* or nutritionist* or radiologist* or manager* or administrator* or leader* or student* or trainee* or intern* or resident*:ti,ab,kw (Word variations have been searched)

#50 Health near/2 provider:ti,ab,kw (Word variations have been searched)

#51 "physical therapist*":ti,ab,kw (Word variations have been searched)

#52 {or #40-#51}

#53 MeSH descriptor: [Education, Medical] explode all trees

#54 MeSH descriptor: [Internship and Residency] explode all trees

#55 MeSH descriptor: [Education, Nursing] explode all trees

#56 MeSH descriptor: [Clinical Clerkship] explode all trees

#57 #29 or #53 or #54 or #55 or #56

#58 #8 and #39 and #52 and #57 Publication Year from 1996 to 2016 (later rerun to include 01-01-2016 to 01-07-2017)

# Embase

Database: Embase <1996 to 2016 February 02> (later rerun to include 03-02-2016 to 01-07-2017)

Search Strategy:

--------------------------------------------------------------------------------

1 exp sex difference/

2 exp gender bias/

3 sexism/

4 feminism/

5 ((sex adj2 difference*) or (gender adj2 bias) or Sexism or Feminism).tw.

6 ((gender or sex) adj2 (sensitiv* or equit* or inequit* or equalit* or inequalit* or budget* or mainstream* or discriminat* or stereotyp* or bias or issue* or factor* or variab* or difference* or conflict* or diversit* or analysis or relation*)).tw.

7 or/1-6

---------------------------------------------------------------------------------------------------------------------------------

8 exp paramedical education/

9 exp medical education/

10 exp curriculum/

11 exp continuing education/

12 exp in service training/

13 exp manpower planning/

14 exp career mobility/

15 exp income/ or exp "salary and fringe benefit"/

16 exp staff training/

17 exp personnel management/ or exp health care personnel management/

18 (curriculum or education or learning or preceptorship* or mentorship* or residenc* or internship* or training* or orientation* or development or recruitment* or retention* or retaining or hiring or planning or management or selection or staffing or scheduling or motivation or promotion or performance).tw.

19 (turnover or (turn adj over) or (career adj mobility) or workload* or (work adj load*)).tw.

20 (salar* or fringe* or incentive* or income* or wage* or (pay adj equit*) or ((sick or matern*) adj leave*) or absenteeism).tw.

21 or/8-20

---------------------------------------------------------------------------------------------------------------------------------22 exp university/

23 exp university hospital/

24 exp hospital/ or exp teaching hospital/

25 health care facility/ or cancer center/ or clinical laboratory/ or community mental health center/ or core laboratory/ or dental facility/ or health center/ or laboratory/ or pharmacy/ or secondary care center/ or tertiary care center/

26 (((medical or health or academic) adj2 (universit* or center* or centre or school* or facilit*)) or hospital*).tw.

27 or/22-26

---------------------------------------------------------------------------------------------------------------------------------28 exp health care personnel/ or exp hospital personnel/ or exp medical personnel/ or exp paramedical personnel/

29 exp medical student/

30 exp physician/

31 (human adj2 (capital or resource*)).tw.

32 (worker* or (work adj force) or workforce or (man adj power) or manpower or personnel or staff).tw.

33 ((health adj care adj provider*) or doctor* or practitioner* or physician* or clinician* or staff or specialist* or surgeon* or nurse* or pharmacist* or dentist* or dieti?ian* or nutritionist* or (medical adj lab adj specialist*) or radiologist* or physical therapist* or manager* or administrator* or leader* or student* or trainee* or intern*).tw.

34 or/28-33

---------------------------------------------------------------------------------------------------------------------------------35 7 and 21 and 27 and 34

***************************

# Grey literature

### OpenGrey

(Sexism OR “Sex factors” OR “Gender sensitivity” OR “Gender discrimination” OR “gender inequity” OR “Gender inequality” OR “gender discrimination”) AND (“medical education” OR curriculum OR training OR “staff development” OR “personnel management” OR retention OR recruitment OR “needs assessment” OR income) AND (“medical centers” OR universities OR “medical school*” OR hospital*) AND (manpower OR personnel OR staff OR worker* OR workforce OR Doctor* OR nurse* OR pharmacist* OR dietitian* OR student*)

### [ProQuest Dissertation and Theses](http://pqdtopen.proquest.com/)

IF(“Gender sensitivity” AND (“personnel management” OR retention OR recruitment) AND (“medical schools” OR hospitals) AND (manpower OR personnel OR staff))
