# Supplementary figures and images for: Gender equity in planning, development and management of human resources for health: a scoping review
Source: Hum Resour Health. 2019 Jul 11;17:52. doi: 10.1186/s12960-019-0391-3 (PMC6625080; doi:10.1186/s12960-019-0391-3)

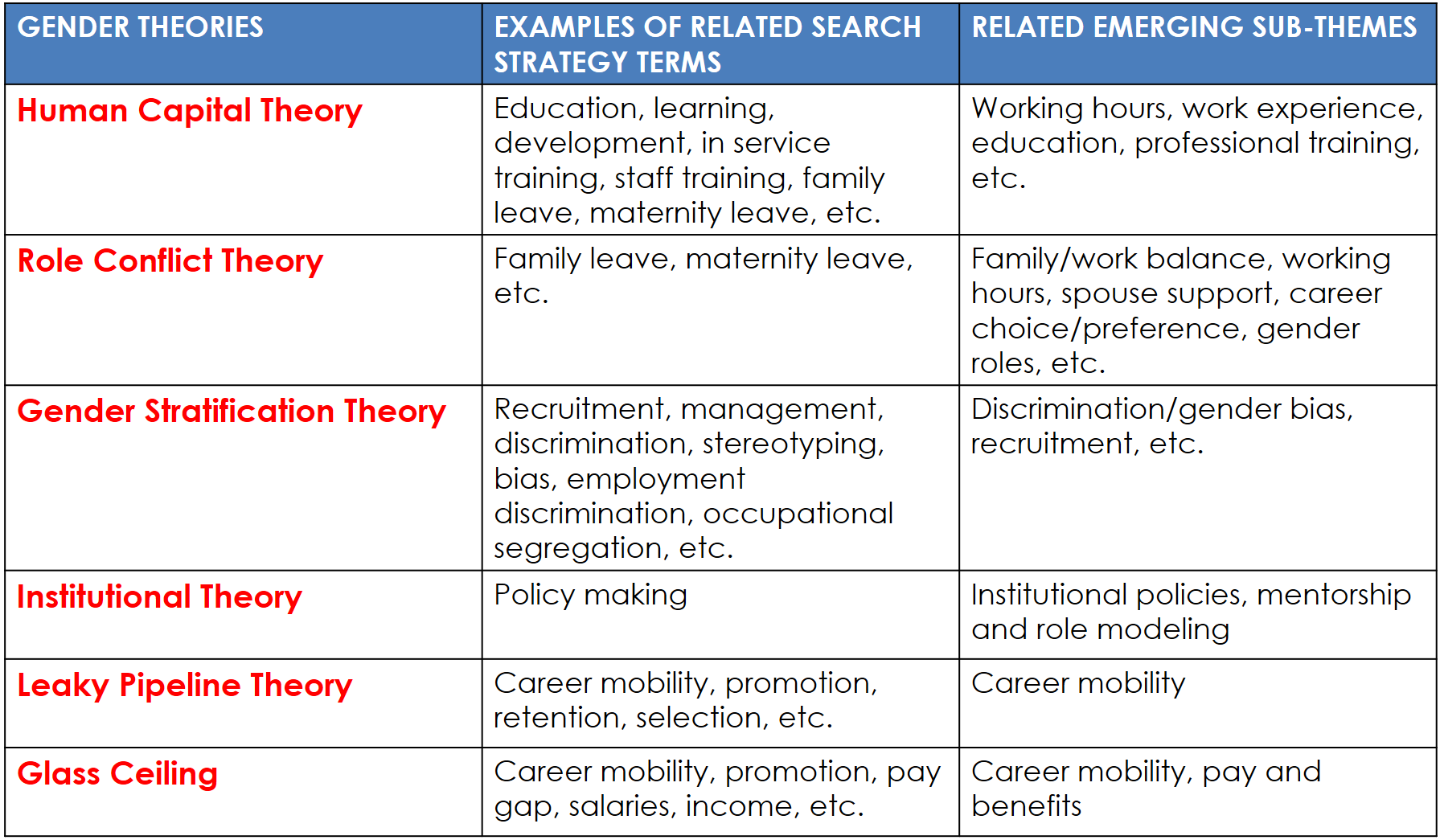

Supplement: Supplementary file 3 — Description of included studies; alignment of gender theories with used search strategy terms and results sub-themes. (PNG 136 kb) [file 12960_2019_391_MOESM3_ESM.png]
